# Whole-Genome Sequencing, Phylogenetic and Genomic Analysis of *Lactiplantibacillus pentosus* L33, a Potential Probiotic Strain Isolated From Fermented Sausages

**DOI:** 10.3389/fmicb.2021.746659

**Published:** 2021-10-26

**Authors:** Odysseas Sotirios Stergiou, Konstantinos Tegopoulos, Despoina Eugenia Kiousi, Margaritis Tsifintaris, Aristotelis C. Papageorgiou, Chrysoula C. Tassou, Nikos Chorianopoulos, Petros Kolovos, Alex Galanis

**Affiliations:** ^1^Department of Molecular Biology and Genetics, Faculty of Health Sciences, Democritus University of Thrace, Alexandroupolis, Greece; ^2^Institute of Technology of Agricultural Products, Hellenic Agricultural Organization DIMITRA, Athens, Greece

**Keywords:** *Lactiplantibacillus pentosus*, whole-genome sequencing, probiotics, comparative genomics, phylogenetic analysis

## Abstract

*Lactobacillus* is a diverse genus that includes species of industrial and biomedical interest. *Lactiplantibacillus pentosus*, formerly known as *Lactobacillus pentosus*, is a recently reclassified species, that contains strains isolated from diverse environmental niches, ranging from fermented products to mammalian gut microbiota. Importantly, several *L. pentosus* strains present health-promoting properties, such as immunomodulatory and antiproliferative activities, and are regarded as potential probiotic strains. In this study, we present the draft genome sequence of the potential probiotic strain *L. pentosus* L33, originally isolated from fermented sausages. Comprehensive bioinformatic analysis and whole-genome annotation were performed to highlight the genetic loci involved in host-microbe interactions and the probiotic phenotype. Consequently, we found that this strain codes for bile salt hydrolases, adhesins and moonlighting proteins, and for Class IIb bacteriocin peptides lacking the GxxxG and GxxxG-like motifs, crucial for their inhibitory activity. Its adhesion ability was also validated *in vitro*, on human cancer cells. Furthermore, *L. pentosus* L33 contains an exopolysaccharide (EPS) biosynthesis cluster, and it does not carry transferable antibiotic resistance genes. Kyoto Encyclopedia of Genes and Genomes (KEGG) pathway and CAZymes analyses showed that *L. pentosus* L33 possesses biosynthetic pathways for seven amino acids, while it can degrade a wide array of carbohydrates. In parallel, Clusters of Orthologous Groups (COGs) and KEGG profiles of *L. pentosus* L33 are similar to those of 26 *L. pentosus* strains, as well as of two well documented *L. plantarum* probiotic strains. Conclusively, *L. pentosus* L33 exhibits good probiotic potential, although further studies are needed to elucidate the extent of its biological properties.

## Introduction

*Lactobacillus* is a diverse genus that includes Gram-positive, facultatively anaerobic, non-spore-forming, hetero-, or homo-fermentative bacteria that inhabit a broad range of nutrient-rich environmental niches (Duar et al., 2017). The species of this genus have been recently reclassified to 25 genera, based on shared ecological and metabolic properties (Zheng et al., 2020). *Lactobacillus* strains can be found as autochthonous or allochthonous, mainly in the mammalian gastrointestinal tract, fresh fruit and vegetable microbiota, as well as in fermented foodstuffs (Inglin et al., 2018). In this context, several strains exhibit great biotechnological interest, due to their fermentation capacity and are being incorporated as starter cultures in a broad range of dairy and non-dairy products (Kok and Hutkins, 2018). Furthermore, specific strains are considered probiotic, meaning that they can confer health benefits to the host, when consumed in adequate quantities (FAO/WHO, 2002). Regarding the proposed positive impact of probiotics on host health, preclinical and clinical studies have shown that they can exhibit antimicrobial (Yu et al., 2015), immunomodulatory (Chondrou et al., 2020), antioxidant (Wu et al., 2019), antiproliferative (Tiptiri-Kourpeti et al., 2016), and even psychobiotic activity (Tian et al., 2020). Today, probiotics are commercially available in supplements or in functional products, comprising a rapidly growing global market, currently worth more than $50 billion, as market reports indicated.^1^

The commercialization of probiotic strains is strictly monitored. Indeed, several guidelines have been set in place for the characterization of novel probiotic strains by the World Health Organization (WHO), the Food and Agriculture Organization of the United Nations (FAO), and the European Food Safety Authority (EFSA) (FAO/WHO, 2002; EFSA, 2018). First, new isolates should be molecularly assigned to a specific taxonomic group. EFSA also requires full genome sequencing and annotation of strains that are intended for biotechnological applications (EFSA, 2018). Importantly, probiotics must be safe for consumption; they should not exhibit hemolytic or virulence activity. Consequently, they should be characterized by either the Food and Drug Administration (FDA) or EFSA with the “Generally Recognized as Safe” (GRAS) or of “Qualified Presumption of Safety” status, respectively (Rodrigo-Torres et al., 2019). Furthermore, probiotic microorganisms should be able to tolerate the gastrointestinal tract conditions; be resistant to low pH, gastric enzymes, and bile acids and, also, adhere to and, at least transiently, colonize the gastrointestinal mucosa (Hill et al., 2014). The proposed health effects of new isolates should be thoroughly explored *in vitro* and *in vivo*, to finally be validated in the clinical setting (FAO/WHO, 2002). Mechanistic studies on host-probiotic interactions have flourished recently, with the advent of multi-omics technologies, facilitating a better understanding of their properties and biological functions (Kiousi et al., 2021).

The introduction of genomics in the microbiology field has restructured the characterization of novel *Lactobacillus* strains as probiotics. As next-generation sequencing platforms are becoming increasingly accessible, the taxonomic and functional characterization of new isolates can be performed with greater accuracy. One of the species that has been reclassified recently is *Lactiplantibacillus pentosus*, formerly known as *Lactobacillus pentosus* (Zheng et al., 2020). The bacteria of this species are mainly associated with environmental samples, such as fruit and vegetable microbiota, however, several strains harbor genes for mammalian host adaptation (Abriouel et al., 2017). Genome mining in *L. pentosus* strains and comparative genomic analysis with the closely related *L. plantarum* species have revealed functional characteristics involved in the probiotic phenotype, such as the presence of genes involved in stress response (Ye et al., 2020), metabolic capacity (Abriouel et al., 2017), adhesion on the intestinal mucosa and bacteriocin production (Maldonado-Barragán et al., 2011).

*L. pentosus* L33 is a LAB (Lactic Acid Bacteria) strain, with desirable probiotic properties, as previously demonstrated in a series of established *in vitro* tests (Pavli et al., 2016). The aim of this study was to further investigate the probiotic potential of the strain by characterizing the genetic basis of the probiotic phenotype. Firstly, whole-genome sequencing was performed to reveal the genomic characteristics of the strain. Then, genome annotation and comparative genomic analysis with other *L. pentosus*, as well as, *L. plantarum* genome sequences were executed to detect strain-specific genes and pinpoint genes of interest. More specifically, the presence of gene clusters involved in the biosynthesis of bacteriocins, adhesion proteins and exopolysaccharides were investigated. Lastly, KEGG pathway and CAZymes analyses were performed to evaluate the metabolic capabilities of *L. pentosus* L33.

## Materials and Methods

### Bacterial Strain, Culture Conditions, and DNA Isolation

*L. pentosus* L33 was originally isolated from fermented sausages (Pavli et al., 2016), and was acquired by the Institute of Technology of Agricultural Products, Hellenic Agricultural Organization DIMITRA (Athens, Greece). It was maintained in de Man, Rogosa, and Sharpe (MRS) broth (Condalab, Madrid, Spain) at 37°C for 16–18 h under anaerobic conditions, prior to DNA extraction. Bacterial cells were collected by centrifugation at 8,000 *g* for 4 min. Total genomic DNA was extracted from the cell pellets using the NucleoSpin^®^ Tissue kit (Macherey-Nagel, Düren, Germany), according to manufacturer’s instructions. DNA purity and quantity were confirmed spectrophotometrically at 260 nm using NanoDrop^®^ ND-1000 UV-Vis Spectrophotometer (Thermo Fisher Scientific, Waltham, MA, United States).

### Whole-Genome Sequencing and Genome Annotation

The genomic DNA of *L. pentosus* L33 was sequenced using Illumina NovaSeq6000 (2 × 151 paired ends) platform. A total of 8,806,648 paired-end reads were obtained. The quality of the reads was estimated using FASTQC (version 0.11.9) (Andrews, 2010), while low-quality reads were removed via Trimmomatic (version 0.39) (Bolger et al., 2014). *De novo* assembly process was executed with SPAdes (version 3.15.1) (Bankevich et al., 2012), selecting the “–careful” option to reduce mismatches and SSPACE_Standard (version 3.0) (Boetzer et al., 2011) with the parameter to filter out contigs with length below 500 base pairs.

Genome annotation was carried out locally, using the Prokaryotic Genome Annotation Pipeline (PGAP) (Tatusova et al., 2016) algorithm with default parameters. EggNOG-mapper (version 2.0) tool from the online EggNOG database (version 5.0) (Huerta-Cepas et al., 2019) was used for functional classification of proteins into COGs. BlastKOALA (version 2.2) was utilized for Kyoto Encyclopedia of Genes and Genomes Orthology (KO) assignment and KEGG mapping of the predicted genes (Kanehisa et al., 2016). Carbohydrate-active enzymes (CAZymes) were searched against the CAZy database (Lombard et al., 2014). Clustered regularly interspaced palindromic repeats (CRISPR) inside the assembly were evaluated using CRISPRDetect (version 2.4) (Biswas et al., 2016). PHAge Search Tool Enhanced Release (PHASTER) (Arndt et al., 2016) was utilized for identification and annotation of putative prophage sequences inside the bacterial assembly. Visualization of the genome assembly was performed by Artemis tool (version 18.1.0) (Carver et al., 2012), while its metrics were calculated with the Quality Assessment Tool (QUAST) (version 5.2.0) (Gurevich et al., 2013).

### Phylogenetic and Comparative Analysis

Average Nucleotide Identity (ANI) analysis was performed on the complete genome assembly, using a python module called Pyani (version 0.2.10) (Pritchard et al., 2016), to verify the taxonomic identity of *L. pentosus* L33. Pangenome analysis of the available *L. pentosus* strains (May 2021), was operated by Roary (version 3.13.0) (Page et al., 2015). The phylogenomic analysis, including 1,000 bootstrap replicates (Maximum Composite Likelihood model), was performed by MEGAX (version 10.1.8) (Kumar et al., 2018). The publicly available online EMBL tool called “Interactive Tree of Life” (iTol) (version 6.1.1) (Letunic and Bork, 2016) was used for phylogenetic tree construction. Strain-specific genes were determined via an in-house Python script.

### Detection of Genetic Elements Associated With Probiotic Characteristics

BAGEL (version 4) (de Jong et al., 2006) was employed for detection and visualization of gene clusters that are involved in bacteriocin biosynthesis. The presence of antibiotic resistance genes was verified by Resistance Gene Identifier (RGI) (version 5.1.1) (Jia et al., 2017). BLAST (basic local alignment search tool) was used for the search of genes that are involved in EPS production, bile salt hydrolysis and cell adhesion.

### Quantitative Adhesion Assay

The assay was performed as described before, with minor modifications (Plessas et al., 2020). Briefly, human colon adenocarcinoma HT-29 cells were seeded in 24-well plates at a density of 40 × 10^4^ cells per well and incubated for 14 days to form a monolayer. The cells were maintained in Roswell Park Memorial Institute (RPMI)-1640 medium enriched with GlutaMAX^TM^, 10% fetal bovine serum (FBS), 100 μg/mL streptomycin and 100 U/mL penicillin (Thermo Fisher Scientific, Waltham, MA, United States) and incubated at 37°C, 5% CO_2_ in a humidified atmosphere. 10^7^ or 10^8^ CFU/mL of viable *L. pentosus* L33 or *L. rhamnosus* GG cells were added to each well. After 4 h of co-incubation at 37°C, the cells were washed with PBS and lysed with 1% Triton X-100 (Sigma-Aldrich, Taufkirchen, Germany). The lysates were serially diluted in Ringer’s solution (Lab M, Lancashire, United Kingdom), plated on 2% MRS agar, and incubated at 37°C, until the formation of visible colonies. For the calculation of adhesion values the following formula was applied: % Adhesion = (V_*B*_/V_*A*_) × 100, where V_*A*_ is the initial viable count of bacteria tested, and V_*B*_ is the viable bacteria count attached on HT-29 cells. Colony forming units per milliliter (CFU/mL) was used as viable count measure that was determined using the formula: CFU/mL = (number of colonies × dilution factor)/volume of culture plate.

## Results

### Genome Features

**FIGURE 1:**
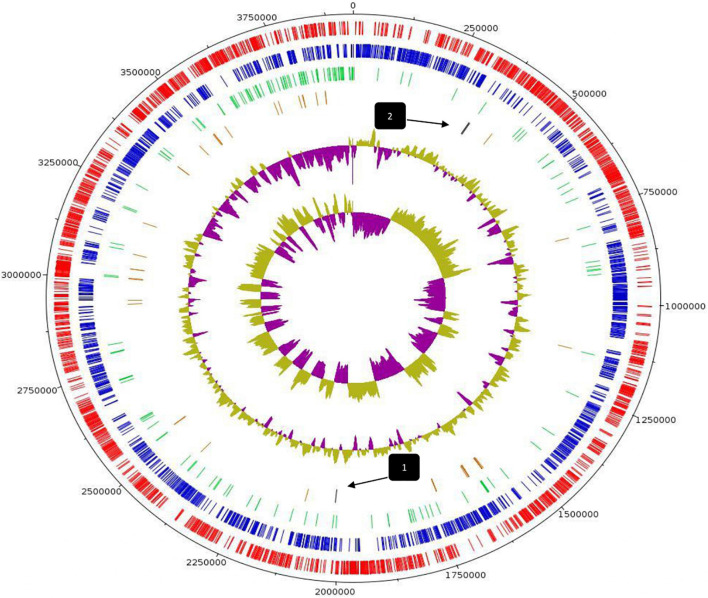
Circular genome map of *L. pentosus* L33. From outer circle to inner, information is displayed as follows: Genome Size (black), Forward strand CDS (red), Reverse strand CDS (blue), Pseudogenes (green), tRNA genes (brown), GC Content, GC Skew. Arrows indicate the position of CRISPR arrays.

Whole-genome sequencing and comprehensive bioinformatic analysis were employed for the investigation of the genomic features of *L. pentosus* L33 (Table 1), ultimately leading to the construction of its genome map (Figure 1). The complete genome of *L. pentosus* L33 has a length of 3,923,201 bp with a GC content of 46.01%. Among the 3,630 predicted genes, 3,429 were found to be protein-coding sequences (CDS). Furthermore, 127 pseudogenes, 58 tRNAs, 6 rRNAs, and 5 ncRNAs were identified. The 58 tRNA encoding sequences correspond to all 20 amino acids (Supplementary Table 1). In addition, 3 clustered regularly interspaced short palindromic repeats (CRISPR) arrays (Supplementary Table 2), as well as 4 intact prophage regions (Supplementary Table 3) were recognized.

**TABLE 1:** *Lactiplantibacillus pentosus* L33 genome features.

**Attribute**	**Values**
Genome size (bp)	3,932,201
GC content (%)	46.01
Total genes	3,643
CDS (protein)	3,423
Pseudogenes	151
tRNA genes	58
rRNA genes	6
ncRNA genes	5

### Phylogenetic Analysis and Unique Genome Characteristics of *Lactiplantibacillus pentosus* L33

**FIGURE 2:**
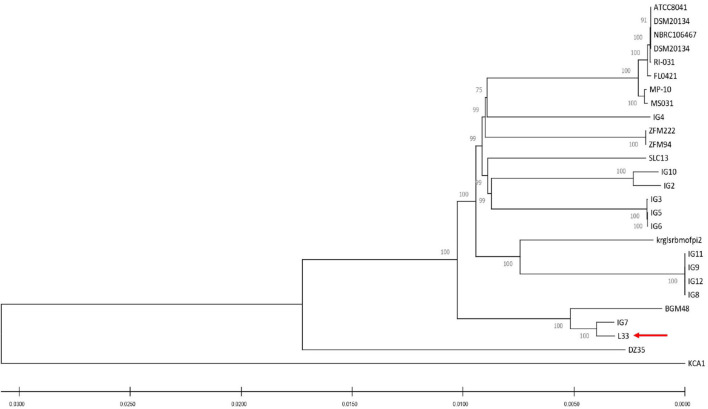
Neighbor-joining phylogenetic tree of *L. pentosus* L33 and 26 *L. pentosus* strains based on orthologous genes found by Roary (version 3.13.0). Values of 1,000 bootstrap replicates calculated by MEGAX (version 10.1.8) are depicted.

**FIGURE 3:**
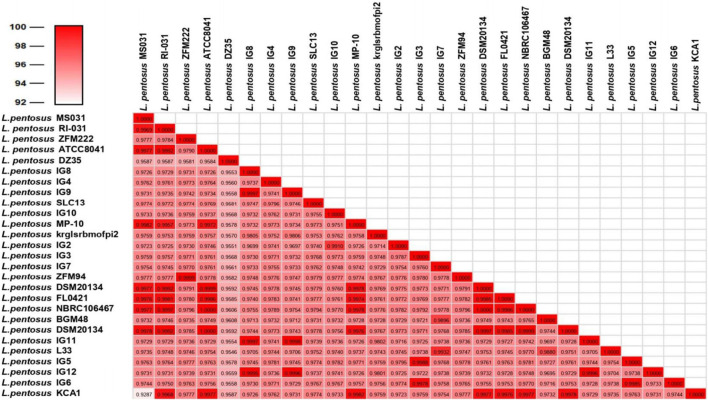
Average Nucleotide Identity scores among all 27 *L. pentosus* strains.

**FIGURE 4:**
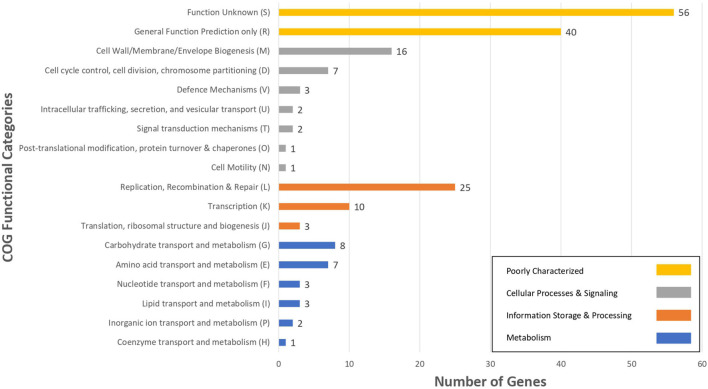
The number of *L. pentosus* L33 strain specific genes, compared to 26 *L. pentosus* strains, assigned to COGs functional categories. Different bar colors represent the further classification of all functional categories into four major classes—Poorly Characterized (Yellow bars), Cellular Processes and Signaling (Gray bars), Information Storage and Processing (Orange bars) and Metabolism (Blue bars).

For the characterization of strain L33, sequencing of the V1-V3 region of 16S rRNA gene, followed by multiplex PCR targeting the recA gene was performed. Strain L33 was assigned to the species of *Lactobacillus pentosus* (Pavli et al., 2016), currently known as *Lactiplantibacillus pentosus* (Zheng et al., 2020). A neighbor-joining phylogenetic tree, including 1,000 bootstrap replications, based on orthologous gene clusters, was built to reveal the exact phylogenetic position of *L. pentosus* L33 within *L. pentosus* species (Figure 2). Moreover, we have used 26 *L. pentosus* strains, 2 well documented *L. plantarum* probiotic strains; *L. plantarum* WCFS1 (van den Nieuwboer et al., 2016) and *L. plantarum* 299v (Nordström et al., 2021), as well as *Staphylococcus aureus* NCTC8325 and *Streptococcus pneumoniae* NCTC11032, as controls (Supplementary Figure 1). The closest evolutionary relatives of *L. pentosus* L33 are *L. pentosus* IG7, which was isolated from the brine of natural Spanish-style green olive fermentation (Calero-Delgado et al., 2018), and *L. pentosus* BGM48, originated from laboratory scale Sicilian-style green olive fermentation (Golomb et al., 2013; Figure 2). Furthermore, when compared to *L. pentosus* L33, ANI analysis found that *L. pentosus* IG7 and *L. pentosus* BGM48 exhibit the greatest ANI scores, with 99.3 and 98.8%, respectively. The full ANI matrix, including all genomes, is presented in Figure 3. Additionally, *L. pentosus* L33, when comparing to 26 *L. pentosus* analyzed genomes, has 243 genes (6.60%) that were found to be strain-specific. The proteins encoded by unique genes were classified into COG functional categories (Figure 4). A total of 190 (78.18%) unique proteins were assigned to 18 COG functional categories. The majority (96 proteins) were categorized as “poorly characterized.”

### Functional Classification

**FIGURE 5:**
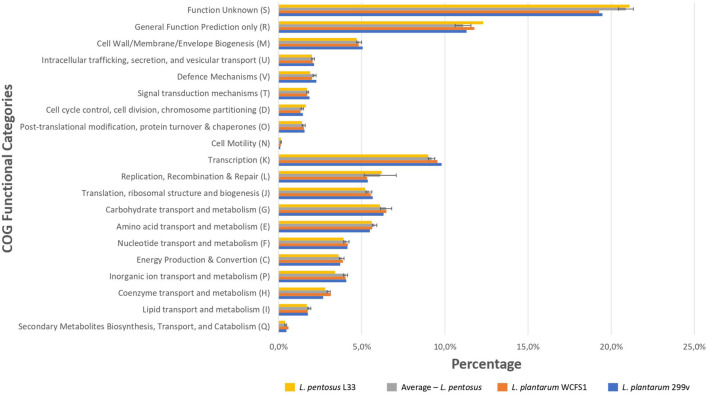
Comparison of the percentage of genes assigned to the COG functional categories of *L. pentosus* L33 (Yellow bars), *L. plantarum* WCFS1 (Orange bars), *L. plantarum* 299v (Blue bars) and of 26 *L. pentosus* strains (Gray bars). We calculated the percentage of genes for each COG functional category, for each one of the 26 *L. pentosus* strains, and depict their average values alongside the standard deviation (Gray bars).

We sought to perform *in silico* functional classification of *L. pentosus* L33 and applied various interconnected approaches to achieve a well-rounded categorization of its genes/CDSs. The COG database is a valuable tool for describing the functional characteristics of newly sequenced genomes, as well as, comparing microbial communities (Galperin et al., 2019). Moreover, KEGG analysis is used to examine the diversity, as well as, the functionality of the proteins. Therefore, we performed a comprehensive analysis and comparison of the COG and KEGG profiles for *L. pentosus* L33, 26 *L. pentosus* strains, *L. plantarum* WCFS1 and *L. plantarum* 299v. The vast majority (94.66%) of the CDSs of *L. pentosus* L33, were allocated to 20 COG functional categories (Figure 5). The category “Function Unknown” was the most abundant (21.1%), followed by “General Function Prediction only” (12.3%), “Transcription” (9.0%), “Replication, Recombination, and Repair” (6.2%), “Carbohydrate transport and metabolism” (6.1%). Furthermore, comparison of the COG profile of *L. pentosus* L33 with the respective COG profiles of the 26 *L. pentosus* strains, *L. plantarum* WCFS1, and *L. plantarum* 299v, highlighted its similarity in respect to the percentage of the genes allocated in each of the COG functional categories (Figure 5 and Supplementary Table 4). The abovementioned similarity is irrelevant of the isolation source of the analyzed strains, since they have been derived from a variety of ecological niches such as meat samples, olive brines, milk products, fermented vegetables, and human intestine etc.

**FIGURE 6:**
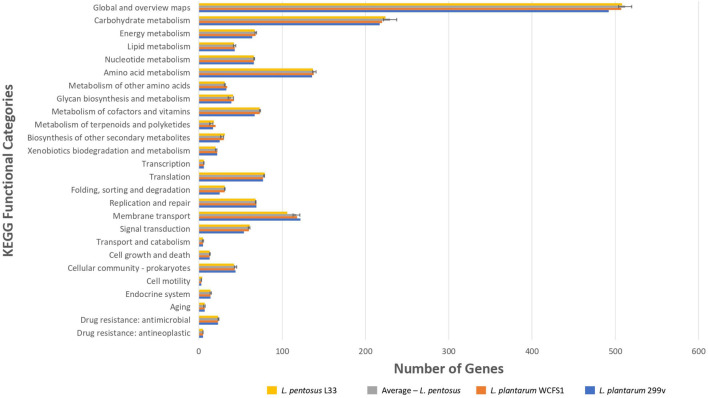
Comparison of the number of genes assigned to the most abundant KEGG functional categories of *L. pentosus* L33 (Yellow bars), *L. plantarum* WCFS1 (Orange bars), *L. plantarum* 299v (Blue bars), and of 26 *L. pentosus* strains (Gray bars). We calculated the number of genes for each KEGG functional category, for each one of the 26 *L. pentosus* strains, and depict their average values alongside the standard deviation (Gray bars).

To unveil the functional characterization of the CDSs of *L. pentosus* L33, we performed KEGG analysis. More precisely, approximately half of the *L. pentosus* L33 CDSs (52.10%) were assigned to 39 KEGG functional categories and 189 pathways. These pathways are mainly involved in the biosynthesis of secondary metabolites (ko: 01110; 180 genes), microbial metabolism in diverse environments (ko: 01120; 100 genes), and biosynthesis of amino acids (ko: 01230; 86 genes). Similarly to COG profiles, the number of genes assigned to each of the KEGG functional categories, is similar between *L. pentosus* L33, the other 26 *L. pentosus* strains, *L. plantarum* WCFS1, and *L. plantarum* 299v (Figure 6 and Supplementary Table 5). In addition, five virulence factors were identified in *L. pentosus* L33, including, a molecular chaperone (Hsp33), a translocase (YidC), two proteins of *Mycobacterium tuberculosis* with poorly defined function (Jag and YidD) (Yu et al., 2011), and a hemolysin iii family protein. However, the functionality of the detected hemolysin remains questionable, due to reports which indicate that *L. pentosus* L33 does not exhibit hemolytic activity *in vitro* (Pavli et al., 2016).

Furthermore, employing CAZymes analysis, we classified the genes into respective CAZymes gene families (Supplementary Table 6). Thus, we showed that the *L. pentosus* L33 genome contains 92 genes, which were categorized into four CAZymes gene classes: 48 glycoside hydrolase (GH) genes, 34 glycosyltransferase (GT) genes, 7 carbohydrate-binding modules (CBMs), 3 carbohydrate esterase (CE) genes.

### Identification of Genes Implicated in the Probiotic Potential of *Lactiplantibacillus pentosus* L33

**FIGURE 7:**
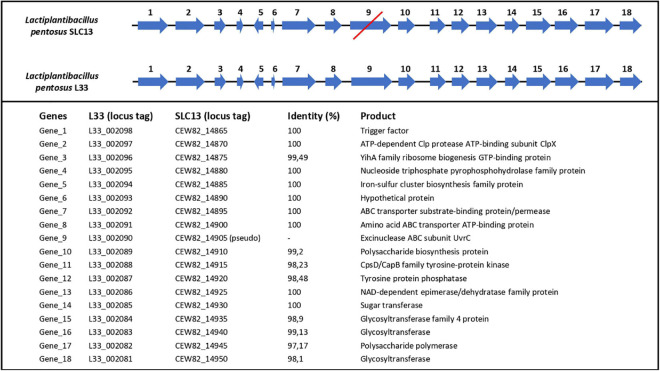
Comparison of length, position and direction of genes comprising the EPS biosynthesis cluster of *L. pentosus* L33 and *L. pentosus* SLC13. Protein identities between the two strains are also displayed. The red line indicates that gene number 9 in *L. pentosus* SLC13 is a pseudogene.

Finally, we performed comparative and comprehensive bioinformatical analysis to analyze in depth the *L. pentosus* L33 genome and locate genes and/or regions endowing a probiotic potential. Prokaryotic Genome Annotation Pipeline (PGAP) predicted that *L. pentosus* L33 contains 4 genes related to bile salt resistance; two bile salt hydrolases and two enzymes that are members of the GCN5-related N-acetyltransferases family (GNAT) (Table 2). Furthermore, RGI showed that the resistome of *L. pentosus* L33 does not contain transferable antibiotic resistance genes. Furthermore, a gene cluster consisting of 18 genes, involved in EPS biosynthesis, was identified during genome annotation. The aforementioned cluster has been, previously, described in the potential probiotic strain *L. pentosus* SLC13 and it is also present in the probiotic strain *L. plantarum* WCFS1 (Huang et al., 2018). Comparison between the EPS gene clusters indicated that the genes carried by *L. pentosus* L33 are homologous to those of strain SLC13, which is a potent exopolysaccharide producing strain (Figure 7; Huang et al., 2018).

**TABLE 2:** List of bile salt resistance genes identified in *L. pentosus* L33.

**Gene ID**	**Length (bp)**	**Product**
L33_000531	1,016	Choloylglycine hydrolase family protein
L33_001135	986	Choloylglycine hydrolase family protein
L33_003442	524	GNAT family N-acetyltransferase
L33_000102	518	GNAT family N-acetyltransferase

**FIGURE 8:**
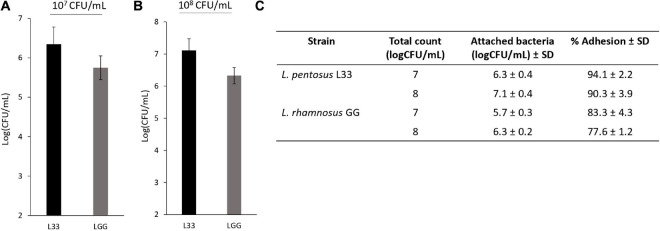
Assessment of the adhesion capacity of *L. pentosus* L33 by quantitative analysis. *L. rhamnosus* GG was used as a reference strain. **(A)** Adhesion capacity of viable cells at a concentration of 10^7^ CFU/mL to HT-29 cells after 4-h co-incubation. **(B)** Adhesion capacity of viable cells at a concentration of 10^8^ CFU/mL to HT-29 cells after 4-h co-incubation. **(C)** Adhesion (%) of attached bacteria to initial added total count. The data presented are the mean ± standard deviation of three independent experiments performed in duplicates.

In addition, *L. pentosus* L33 contains 3 mucus-binding domain containing proteins and 2 proteins with fibronectin-binding domains along with NFACT domains (Table 3). Furthermore, 6 surface proteins carrying LPxTG cell wall anchored motifs were identified (Table 3). Moreover, moonlighting proteins with adhesin-like activity, elongation factor Tu, chaperonin GroEL, and co-chaperone GroES, are also present in the genome of *L. pentosus* L33 (Table 3). Notably, the adhesion capacity of *L. pentosus* L33 was validated *in vitro* utilizing HT-29 cells. Importantly, the strain exhibited similar adhesion capacity to that of the reference strain, *L. rhamnosus* GG (Figure 8).

**TABLE 3:** List of proteins involved in the adhesion of *L. pentosus* L33 on host cells.

**Gene ID**	**Length (bp)**	**Product**
L33_000934	3,371	MucBP domain-containing protein
L33_002751	2,408	MucBP domain-containing protein
L33_001971	1,043	MucBP domain-containing protein
L33_003298	1,706	NFACT family protein
L33_001396	881	NFACT family protein
L33_002099	1,187	Elongation factor Tu
L33_001213	284	Co-chaperone GroS
L33_001214	1,625	Chaperonin GroEL
L33_002651	941	Zinc ABC transporter substrate-binding protein
L33_001981	1,328	Phosphopyruvate hydratase
L33_000881	434	LPXTG cell wall anchor domain-containing protein
L33_001642	368	LPXTG cell wall anchor domain-containing protein
L33_000299	2,507	LPXTG cell wall anchor domain-containing protein
L33_000320	1,409	LPXTG cell wall anchor domain-containing protein
L33_000373	2,345	LPXTG cell wall anchor domain-containing protein
L33_001378	1,334	LPXTG cell wall anchor domain-containing protein

Concerning the antimicrobial activity of the studied strain, *L. pentosus* L33 encodes for a class IIb bacteriocin, which is homologous to plantaricin NC8 αβ (Bengtsson et al., 2020). Class IIb bacteriocins consist of two peptides, which mediate their action by the interaction of their GxxxG and GxxxG-like (SxxxS and GxxxS) motifs with the membrane of the target pathogen (Maldonado et al., 2003; Bengtsson et al., 2020). The peptides coded by this strain lack a GxxxG-like motif and as a result, the functionality of the final product might be seriously affected (Supplementary Figure 2).

## Discussion

In this study, we present the draft genome sequence of *Lactiplantibacillus pentosus* L33, a strain isolated from traditional meat products (Pavli et al., 2016). The genome of this strain consists of a circular chromosome; with no plasmid sequences detected. The complete genomic length (3,923,201 bp) and GC content (46.01%) of *L. pentosus* L33 were found to be similar to that of other *L. pentosus* strains, such as the closely related *L. pentosus* IG7 (3,802,404 bp, GC content: 45.79%, Accession: GCA_002993395.1) (Calero-Delgado et al., 2018), and the potential probiotic strain *L. pentosus* MP-10 (3,698,214 bp, GC content: 46.00%, Accession: GCA_900092635.1) (Abriouel et al., 2017). The genomic size and GC content of strains could be indicative of their lifestyle and preferred environmental niche. Strains of the *Lactobacillus sensu lato* that are free-living or nomadic usually possess a larger genome with an approximate length of 3–4 Mb, whereas host-adapted strains have a drastically smaller genome due to gene loss (Duar et al., 2017). The genetic traits that can be affected by this event are clusters for amino acid synthesis, and genes involved in metabolism regulation (Zheng et al., 2015). In this study, we found that the genes of *L. pentosus* L33 are involved in the complete biosynthesis of seven amino acids (Supplementary Table 7 and Supplementary Figures 3–8) and encode part of the required proteins necessary for the biosynthesis of the rest 13 amino acids. Interestingly, comprising of 100 genes, the “microbial metabolism in diverse environments” pathway (ko: 01120) was the second most common. The modules of this pathway include carbohydrate, methane, nitrogen, co-factor, and vitamin metabolism, among others. Concerning carbohydrate metabolism, CAZymes analysis showed that *L. pentosus* L33 does, indeed, code for enzymes involved in the synthesis and degradation of a broad array of simple and complex carbohydrates, such as glucose, galactose, mannose, trehalose, xylose, chitin, and cellulose. Additionally, it codes for galactose-, lactose-, starch-, and glycogen- binding modules that facilitate the catalytic activity of hydrolases (Boraston et al., 2004). These findings suggest that *L. pentosus* L33 may be able to inhabit a broad range of environmental niches.

Concerning the functional properties of this strain, *L. pentosus* L33 has presented desirable attributes in a previous *in vitro* study, where a total of 48 *Lactobacillus* strains were assessed for their susceptibility to common antibiotics, hemolytic activity, tolerance to gastrointestinal conditions, and antimicrobial properties (Pavli et al., 2016). It was found that *L. pentosus* L33 exhibited good tolerance to bile salts, that was not accompanied by bile hydrolase activity. Interestingly, in the present study, we detected two coding sequences for bile salt hydrolases (Table 2); however, their functionality is questioned based on the *in vitro* findings. Nevertheless, it is important to note that bile salt resistance is a complex phenotype that can be mediated by several mechanisms, such as bile-efflux systems, changes in EPS and S-layer protein production (Ruiz et al., 2013). This character should be explored in greater depth in future studies.

In this study, we found that this strain does not carry transferable antibiotic resistance genes. In fact, with the exception of vancomycin, *L. pentosus* L33 was not able to survive treatments with common antibiotics (Pavli et al., 2016). Vancomycin resistance in *Lactobacillus* strains is considered to be intrinsic (Guo et al., 2017); therefore, it is no surprise that *L. pentosus* L33 presents resistance to vancomycin. Consequently, the demonstrated resistance does not raise any safety concerns, as there is no implication of horizontal gene transfer (Shao et al., 2015). The mode of action of this antibiotic involves its interaction with peptidoglycan precursors, leading to the inhibition of cell wall synthesis. More specifically, vancomycin binds to the D-alanine/D-alanine terminus of the muramyl pentapeptide and inhibits the polymerization of the peptidoglycan precursor. In this context, we found that *L. pentosus* L33 possesses a gene (VanX) encoding a D-ala-D-ala dipeptidase which hydrolyzes D-alanine/D-alanine residues (Liu et al., 2009). Moreover, in several LAB species, the D-alanine residue located at the end of the pentapeptide is substituted by D-lactate or D-serine and thus blocking vancomycin binding (Delcour et al., 1999). KEGG analysis showed that *L. pentosus* L33 encodes a Ddl ligase, responsible for the D-alanine to D-lactate substitution in Lactobacilli (Tuyarum et al., 2021). Furthermore, there are reports that indicate that many LAB genera exhibit intrinsic resistance to other antibiotics, such as bacitracin, kanamycin, teicoplanin, and quinolones (Imperial and Ibana, 2016). Additionally, it should be noted, that transfer of the vancomycin resistance cluster from *Enterobacteriaceae* to commercial probiotic strains has been reported *in vitro* and *in vivo*, during transit in the murine gastrointestinal tract (Mater et al., 2008).

The cellular surface of Lactobacilli is decorated by a plethora of cell surface proteins that can interact with host receptors and give rise to a variety of probiotic effects (Teame et al., 2020). Indeed, probiotics can interact with the gastrointestinal mucosa of mammalian hosts utilizing pilli, mucin-, and fibronectin- binding proteins, as well as surface-layer (S-layer) proteins (Siciliano et al., 2019). These interactions are necessary for the transient attachment of ingested probiotics in the intestinal mucosa, while they can also facilitate important probiotic functions, including antimicrobial (Tytgat et al., 2016) and immunomodulatory activity (Monteagudo-Mera et al., 2019). In this study, we found that *L. pentosus* L33 carries mucus- and fibronectin- binding proteins (Table 3), however, it does not encode for spaCBA pilli, commonly found in other LAB strains, such as *L. rhamnosus* GG (Reunanen et al., 2012). The adhesins are covalently anchored to the peptidoglycan layer by a C-terminal Leu-Pro-any-Thr-Gly (LPxTG) motif, which is, also, used for their identification *in silico* (Siegel et al., 2017). Moreover, cytoplasmic proteins that participate in important housekeeping functions such as carbohydrate metabolism, translation regulation and protein folding, can be found, anchorless, in the cellular envelope acting as adhesins (Celebioglu et al., 2017). These multifunctional proteins, also known as moonlighting proteins, have been identified in animals, plants, yeast and bacteria. *L. pentosus* L33 encodes some of these proteins; elongation factor Tu (EF-Tu), chaperonin GroEL, and co-chaperonin GroES. Previous reports have shown that *L. plantarum* and *L. pentosus* strains utilize EF-Tu (Choudhary et al., 2019) and GroEL (Calasso et al., 2013) for the adhesion on the intestinal epithelium. The adhesion capacity of the strain was further validated *in vitro.* We showed that *L. pentosus* L33 can efficiently adhere to HT-29 cells, exhibiting similar behavior to *L. rhamnosus* GG, a reference strain, whose capacity to attach to and colonize the gastrointestinal mucosa has been previously described (Chondrou et al., 2018; Pagnini et al., 2018). Further studies are required to evaluate this finding and elucidate its contribution to probiotic efficacy.

Furthermore, we report that the *L. pentosus* L33 genome includes five virulence factors. Hemolysin iii family protein is very common among *Lactiplantibacillus* genomes, including the probiotic strains *L. plantarum* 299v and *L. plantarum* ST-III. The abovementioned strains have an established safety profile, and they are widely used as probiotics (Chokesajjawatee et al., 2020). Heat shock protein 33 (Hsp33) is a redox-regulated molecular chaperone that binds to unfolded proteins and prevents protein aggregation (Winter et al., 2005). YidC gene encodes a translocase that regulates respiratory metabolism in *Mycobacterium tuberculosis* (Thakur et al., 2016). YidD and Jag belong to the same gene cluster along with YidC, but their function remains unclear (Yu et al., 2011). Nevertheless, the impact of these factors in the safety profile of *L. pentosus* L33 has to be further examined.

The probiotic character has also been linked to EPS biosynthesis, as it is well established that EPS play a key role in the dynamic interaction of bacteria with their environment (Angelin and Kavitha, 2020). EPS can be found loosely attached to the cell surface or excreted in the growth medium of the producer strain, while the yield of production can fluctuate based on growth conditions. In addition, the produced exopolysaccharides can vary in terms of monosaccharide constitution, charge, linkage, and existence of repeated sidechains (Caggianiello et al., 2016). EPS can facilitate niche adaptation, as they promote auto-aggregation (Aslim et al., 2007), attachment to abiotic or biotic surfaces and biofilm formation (Castro-Bravo et al., 2018). Furthermore, there are several physiological functions attributed to EPS such as anti-inflammatory, antioxidant, antiviral, and antiproliferative activity (Nguyen et al., 2020). Lastly, the production of EPS in high concentrations can alter the organoleptic characteristics of fermented products (Ale et al., 2020). In this study, we found an EPS biosynthesis cluster, homologous to that of *L. pentosus* SLC13, a LAB strain known for its capacity to produce high yields of EPS (Huang et al., 2018). In this context, the EPS fraction of *L. pentosus* L33, is currently being studied for its antimicrobial and antibiofilm potential.

Our analysis showed that *L. pentosus* L33 does not code for functional bacteriocins, due to the lack of motifs crucial for their inhibitory action. This finding agrees with previous *in vitro* studies, where no bacteriocin-like activity was detected (Pavli et al., 2016). However, probiotics can exert antimicrobial effects through various mechanisms. such as competition for nutrients, inhibition of pathogen adhesion (Walsham et al., 2016) and immune system stimulation (Tuo et al., 2018). Moreover, they can produce other inhibitory compounds, fatty acids, hydrogen peroxide, ethanol (Chen et al., 2019) and biosurfactants (Sharma and Saharan, 2016), or induce indirect antimicrobial effects by lowering of intestinal pH, due to production of high amounts of lactic and acetic acids. Thus, ongoing studies focus on the potential of this strain to interfere with proliferation and biofilm formation of clinically relevant strains, such as *Staphylococcus aureus, Salmonella enteritidis*, and *Escherichia coli*, by alternative mechanisms to bacteriocin production.

Conclusively, these findings in combination with previous *in vitro* work, support that *L. pentosus* L33 is a good probiotic candidate. This strain fulfills the main criteria for probiotic selection; tolerance to gastrointestinal tract conditions, susceptibility to common antibiotics and γ-hemolytic activity. In the present study, we introduced new traits that add to the characterization of *L. pentosus* L33 as a novel probiotic strain, the capacity to produce adhesins and exopolysaccharides. Whole-genome sequencing and comprehensive bioinformatic analysis facilitate targeted laboratory validation of traits of newly isolated strains, streamlining their characterization as probiotic. In this context, future studies will demonstrate the *in situ* performance of *L. pentosus* L33 strain as a starter/adjunct culture for the production of fermented dry meat products (as this strain was previously isolated from fermented sausages), to signify its effectiveness for application in sausage manufacturing. Additionally, future researches will explore the *L. pentosus* L33-host interactome, and especially gut colonization mechanisms. Overall, *L. pentosus* L33 exhibits a great interest as a potential probiotic strain and forthcoming studies will further unravel its characteristics *in vitro*, *in vivo*, and *in situ.*

## Data Availability Statement

The datasets presented in this study have been submitted to DDBJ/ENA/GenBank under the accession number JAHKRU000000000. The version described in this manuscript is the JAHKRU010000000.

## Author Contributions

AP, NC, PK, and AG designed the study. OS, KT, DK, and MT performed genome analysis and participated in the writing of the manuscript. AP, CT, NC, PK, and AG contributed to editing and critical reviewing of the manuscript. CT and NC took charge of the resources. All authors had read and approved the final manuscript.

## Conflict of Interest

The authors declare that the research was conducted in the absence of any commercial or financial relationships that could be construed as a potential conflict of interest.

## Publisher’s Note

All claims expressed in this article are solely those of the authors and do not necessarily represent those of their affiliated organizations, or those of the publisher, the editors and the reviewers. Any product that may be evaluated in this article, or claim that may be made by its manufacturer, is not guaranteed or endorsed by the publisher.

## Footnotes

1 https://www.grandviewresearch.com/industry-analysis/probiotics-market (Grand View Research, 2021).

## References

[B1] Abriouel H. Pérez Montoro B. Casimiro-Soriguer C. S. Pérez Pulido A. J. Knapp C. W. Caballero Gómez N. (2017). Insight into potential probiotic markers predicted in *Lactobacillus pentosus* MP-10 genome sequence. *Front. Microbiol*. 22:891. 10.3389/fmicb.2017.00891 28588563 PMC5439011

[B2] Ale E. C. Rojas M. F. Reinheimer J. A. Binetti A. G. (2020). *Lactobacillus* fermentum: Could EPS production ability be responsible for functional properties? *Food Microbiol*. 90:103465. 10.1016/j.fm.2020.103465 32336376

[B3] Andrews S. (2010). *FastQC: a Quality Control Tool for High Throughput Sequence Data.* Available online at: https://www.bioinformatics.babraham.ac.uk/projects/fastqc (accessed May 2, 2021).

[B4] Angelin J. Kavitha M. (2020). Exopolysaccharides from probiotic bacteria and their health potential. *Int. J. Biol. Macromol.* 162 853–865. 10.1016/j.ijbiomac.2020.06.190 32585269 PMC7308007

[B5] Arndt D. Grant J. R. Marcu A. Sajed T. Pon A. Liang Y. (2016). PHASTER: a better, faster version of the PHAST phage search tool. *Nucleic Acids Res.* 44 W16–W21. 10.1093/nar/gkw387 27141966 PMC4987931

[B6] Aslim B. Onal D. Beyatli Y. (2007). Factors influencing autoaggregation and aggregation of *Lactobacillus* delbrueckii subsp. bulgaricus isolated from handmade yogurt. *J. Food Prot.* 70 223–227. 10.4315/0362-028x-70.1.223 17265886

[B7] Bankevich A. Nurk S. Antipov D. Gurevich A. A. Dvorkin M. Kulikov A. S. (2012). SPAdes: a new genome assembly algorithm and its applications to single-cell sequencing. *J. Comp. Biol.* 19 455–477. 10.1089/cmb.2012.0021 22506599 PMC3342519

[B8] Bengtsson T. Selegård R. Musa A. Hultenby K. Utterström J. Sivlér P. (2020). Plantaricin NC8 αβ exerts potent antimicrobial activity against *Staphylococcus* spp. and enhances the effects of antibiotics. *Sci. Rep.* 10:3580. 10.1038/s41598-020-60570-w 32107445 PMC7046733

[B9] Biswas A. Staals R. H. Morales S. E. Fineran P. C. Brown C. M. (2016). CRISPRDetect: A flexible algorithm to define CRISPR arrays. *BMC Genom.* 17:356. 10.1186/s12864-016-2627-0 27184979 PMC4869251

[B10] Boetzer M. Henkel C. V. Jansen H. J. Butler D. Pirovano W. (2011). Scaffolding pre-assembled contigs using SSPACE. *Bioinformatics* 27 578–579. 10.1093/bioinformatics/btq683 21149342

[B11] Bolger A. M. Lohse M. Usadel B. (2014). Trimmomatic: A flexible trimmer for Illumina sequence data. *Bioinformatics* 30 2114–2120. 10.1093/bioinformatics/btu170 24695404 PMC4103590

[B12] Boraston A. B. Bolam D. N. Gilbert H. J. Davies G. J. (2004). Carbohydrate-binding modules: fine-tuning polysaccharide recognition. *Biochem. J.* 382 769–781. 10.1042/BJ20040892 15214846 PMC1133952

[B13] Caggianiello G. Kleerebezem M. Spano G. (2016). Exopolysaccharides produced by lactic acid bacteria: from health-promoting benefits to stress tolerance mechanisms. *Appl. Microbiol. Biotechnol*. 100 3877–3886. 10.1007/s00253-016-7471-2 27020288

[B14] Calasso M. Di Cagno R. De Angelis M. Campanella D. Minervini F. Gobbetti M. (2013). Effects of the peptide pheromone plantaricin A and cocultivation with *Lactobacillus sanfranciscensis* DPPMA174 on the exoproteome and the adhesion capacity of *Lactobacillus plantarum* DC400. *Appl. Environ. Microbiol.* 79 2657–2669. 10.1128/AEM.03625-12 23396346 PMC3623163

[B15] Calero-Delgado B. Martín-Platero A. M. Pérez-Pulido A. J. Benítez-Cabello A. Casimiro-Soriguer C. S. Martínez-Bueno M. (2018). Draft genome sequences of six *Lactobacillus pentosus* strains isolated from brines of traditionally fermented spanish-style green table olives. *Genome Announc.* 6:e379–18. 10.1128/genomeA.00379-18 29724847 PMC5940955

[B16] Carver T. Harris S. R. Berriman M. Parkhill J. McQuillan J. A. (2012). Artemis: an integrated platform for visualization and analysis of high-throughput sequence-based experimental data. *Bioinformatics* 28 464–469. 10.1093/bioinformatics/btr703 22199388 PMC3278759

[B17] Castro-Bravo N. Wells J. M. Margolles A. Ruas-Madiedo P. (2018). Interactions of surface exopolysaccharides from *Bifidobacterium* and *Lactobacillus* within the intestinal environment. *Front. Microbiol*. 9:2426. 10.3389/fmicb.2018.02426 30364185 PMC6193118

[B18] Celebioglu H. U. Olesen S. V. Prehn K. Lahtinen S. J. Brix S. Hachem M. A. (2017). Data regarding the growth of *Lactobacillus acidophilus* NCFM on different carbohydrates and recombinant production of elongation factor G and pyruvate kinase. *Data Brief* 14 118–122. 10.1016/j.dib.2017.07.021 28861445 PMC5567391

[B19] Chen C. C. Lai C. C. Huang H. L. Huang W. Y. Toh H. S. Weng T. C. (2019). Antimicrobial activity of *Lactobacillus* species against carbapenem-resistant *Enterobacteriaceae*. *Front. Microbiol.* 10:789. 10.3389/fmicb.2019.00789 31057508 PMC6482263

[B20] Chokesajjawatee N. Santiyanont P. Chantarasakha K. Kocharin K. Thammarongtham C. Lertampaiporn S. (2020). Safety assessment of a nham starter culture *Lactobacillus plantarum* BCC9546 via whole-genome analysis. *Sci. Rep.* 10 1–12. 10.1038/s41598-020-66857-2 32581273 PMC7314741

[B21] Chondrou P. Karapetsas A. Kiousi D. E. Tsela D. Tiptiri-Kourpeti A. Anestopoulos I. (2018). Lactobacillus paracasei K5 displays adhesion, anti-proliferative activity and apoptotic effects in human colon cancer cells. *Benef. Microb*. 9, 975–983. 10.3920/BM2017.0183 30353740

[B22] Chondrou P. Karapetsas A. Kiousi D. E. Vasileiadis S. Ypsilantis P. Botaitis S. (2020). Assessment of the immunomodulatory properties of the probiotic strain *Lactobacillus paracasei* K5 in vitro and in vivo. *Microorganisms* 8:709. 10.3390/microorganisms8050709 32403327 PMC7284587

[B23] Choudhary J. Dubey R. C. Sengar G. Dheeman S. (2019). Evaluation of probiotic potential and safety assessment of *Lactobacillus pentosus* MMP4 isolated from Mare’s lactation. *Probiot. Antimicrob.* Proteins 11 403–412. 10.1007/s12602-018-9431-x 29846884

[B24] de Jong A. van Hijum S. A. Bijlsma J. J. Kok J. Kuipers O. P. (2006). BAGEL: a web-based bacteriocin genome mining tool. *Nucleic Acids Res.* 34 W273–W279. 10.1093/nar/gkl237 16845009 PMC1538908

[B25] Delcour J. Ferain T. Deghorain M. Palumbo E. Hols P. (1999). The biosynthesis and functionality of the cell-wall of lactic acid bacteria. *Anton. Leeuw.* 76 159–184. 10532377

[B26] Duar R. M. Lin X. B. Zheng J. Martino M. E. Grenier T. Pérez-Muñoz M. E. (2017). Lifestyles in transition: evolution and natural history of the genus *Lactobacillus*. *FEMS Microbiol. Rev.* 41 S27–S48. 10.1093/femsre/fux030 28673043

[B27] EFSA (2018). Guidance on the characterisation of microorganisms used as feed additives or 30 as production organisms. *EFSA J.* 16:5206. 10.2903/j.efsa.2018.5206 32625840 PMC7009341

[B28] FAO/WHO (2002). *Guidelines for the Evaluation of Probiotics in Food.* Available online at: https://www.who.int/foodsafety/fs_management/en/probiotic_guidelines.pdf (accessed May 2, 2021).

[B29] Galperin M. Y. Kristensen D. M. Makarova K. S. Wolf Y. I. Koonin E. V. (2019). Microbial genome analysis: the COG approach. *Brief. Bioinform.* 20 1063–1070. 10.1093/bib/bbx117 28968633 PMC6781585

[B30] Golomb B. L. Morales V. Jung A. Yau B. Boundy-Mills K. L. Marco M. L. (2013). Effects of pectinolytic yeast on the microbial composition and spoilage of olive fermentations. *Food Microbiol.* 33 97–106. 23122507 10.1016/j.fm.2012.09.004

[B31] Grand View Research (2021). *Probiotics Market Size, Share and Trends Analysis Report by Product (Food and Beverages, Dietary Supplements), by Ingredient (Bacteria, Yeast), by End Use (Human, Animal), by Distribution Channel, and Segment Forecasts, 2021 – 2028.* San Francisco, CA: Grand View Research.

[B32] Guo H. Pan L. Li L. Lu J. Kwok L. Menghe B. (2017). Characterization of antibiotic resistance genes from *Lactobacillus* isolated from traditional dairy products. *J. Food Sci*. 82 724–730. 10.1111/1750-3841.13645 28182844

[B33] Gurevich A. Saveliev V. Vyahhi N. Tesler G. (2013). QUAST: quality assessment tool for genome assemblies. *Bioinformatics* 29 1072–1075. 10.1093/bioinformatics/btt086 23422339 PMC3624806

[B34] Hill C. Guarner F. Reid G. Gibson G. R. Merenstein D. J. Pot B. (2014). Expert consensus document. The International Scientific Association for Probiotics and Prebiotics consensus statement on the scope and appropriate use of the term probiotic. *Nat. Rev. Gastroenterol. Hepatol.* 11 506–514. 10.1038/nrgastro.2014.66 24912386

[B35] Huang M. L. Huang J. Y. Kao C. Y. Fang T. J. (2018). Complete genome sequence of *Lactobacillus pentosus* SLC13, isolated from mustard pickles, a potential probiotic strain with antimicrobial activity against foodborne pathogenic microorganisms. *Gut. Pathog*. 10:1. 29375672 10.1186/s13099-018-0228-y PMC5774169

[B36] Huerta-Cepas J. Szklarczyk D. Heller D. Hernández-Plaza A. Forslund S. K. Cook H. (2019). eggNOG 5.0: a hierarchical, functionally and phylogenetically annotated orthology resource based on 5090 organisms and 2502 viruses. *Nucleic Acids Res*. 47 D309–D314. 10.1093/nar/gky1085 30418610 PMC6324079

[B37] Imperial I. C. V. J. Ibana J. A. (2016). Addressing the antibiotic resistance problem with probiotics: Reducing the risk of its double-edged sword effect. *Front. Microbiol.* 7:1983. 10.3389/fmicb.2016.01983 28018315 PMC5156686

[B38] Inglin R. C. Meile L. Stevens M. J. A. (2018). Clustering of Pan- and Core-genome of *Lactobacillus* provides novel evolutionary insights for differentiation. *BMC Genom.* 19:284. 10.1186/s12864-018-4601-5 29690879 PMC5937832

[B39] Jia B. Raphenya A. R. Alcock B. Waglechner N. Guo P. Tsang K. K. (2017). CARD 2017: expansion and model-centric curation of the comprehensive antibiotic resistance database. *Nucleic Acids Res.* 45 D566–D573. 10.1093/nar/gkw1004 27789705 PMC5210516

[B40] Kanehisa M. Sato Y. Morishima K. (2016). BlastKOALA and GhostKOALA: KEGG tools for functional characterization of genome and metagenome sequences. *J. Mol. Biol.* 428 726–731. 10.1016/j.jmb.2015.11.006 26585406

[B41] Kiousi D. E. Rathosi M. Tsifintaris M. Chondrou P. Galanis A. (2021). Pro-biomics: Omics technologies to unravel the role of probiotics in health and disease. *Adv. Nutr.* 12 1802–1820. 10.1093/advances/nmab014 33626128 PMC8483974

[B42] Kok C. R. Hutkins R. (2018). Yogurt and other fermented foods as sources of health-promoting bacteria. *Nutr. Rev*. 76 4–15. 10.1093/nutrit/nuy056 30452699

[B43] Kumar S. Stecher G. Li M. Knyaz C. Tamura K. (2018). MEGA X: Molecular Evolutionary Genetics Analysis across computing platforms. *Mol. Biol. Evol.* 35 1547–1549. 10.1093/molbev/msy096 29722887 PMC5967553

[B44] Letunic I. Bork P. (2016). Interactive tree of life (iTOL) v3: an online tool for the display and annotation of phylogenetic and other trees. *Nucleic Acids Res.* 44 W242–W245. 10.1093/nar/gkw290 27095192 PMC4987883

[B45] Liu C. Zhang Z. Y. Dong K. Yuan J. P. Guo X. K. (2009). Antibiotic resistance of probiotic strains of lactic acid bacteria isolated from marketed foods and drugs. *Biomed. Environ. Sci.* 22 401–412. 10.1016/S0895-3988(10)60018-9 20163065

[B46] Lombard V. Golaconda Ramulu H. Drula E. Coutinho P. M. Henrissat B. (2014). The carbohydrate-active enzymes database (CAZy) in 2013. *Nucleic Acids Res.* 42 D490–D495. 10.1093/nar/gkt1178 24270786 PMC3965031

[B47] Maldonado A. Ruiz-Barba J. L. Jiménez-Díaz R. (2003). Purification and genetic characterization of plantaricin NC8, a novel coculture-inducible two-peptide bacteriocin from *Lactobacillus plantarum* NC8. *Appl. Environ. Microbiol.* 69 383–389. 10.1128/AEM.69.1.383-389.2003 12514019 PMC152457

[B48] Maldonado-Barragán A. Caballero-Guerrero B. Lucena-Padrós H. Ruiz-Barba J. L. (2011). Genome sequence of *Lactobacillus pentosus* IG1, a strain isolated from spanish-style green olive fermentations. *J. Bacteriol.* 193:5605. 10.1128/JB.05736-11 21914902 PMC3187417

[B49] Mater D. D. Langella P. Corthier G. Flores M. J. (2008). A probiotic *Lactobacillus* strain can acquire vancomycin resistance during digestive transit in mice. *J. Mol. Microbiol. Biotechnol.* 14 123–127. 10.1159/000106091 17957119

[B50] Monteagudo-Mera A. Rastall R. A. Gibson G. R. Charalampopoulos D. Chatzifragkou A. (2019). Adhesion mechanisms mediated by probiotics and prebiotics and their potential impact on human health. *Appl. Microbiol. Biotechnol.* 103 6463–6472. 10.1007/s00253-019-09978-7 31267231 PMC6667406

[B51] Nguyen P. T. Nguyen T. T. Bui D. C. Hong P. T. Hoang Q. K. Nguyen H. T. (2020). Exopolysaccharide production by lactic acid bacteria: the manipulation of environmental stresses for industrial applications. *AIMS Microbiol.* 6 451–469. 10.3934/microbiol.2020027 33364538 PMC7755584

[B52] Nordström E. A. Teixeira C. Montelius C. Jeppsson B. Larsson N. (2021). *Lactiplantibacillus plantarum* 299v (LP299V^®^): three decades of research. *Benef. Microbes* 12 441–465. 10.3920/BM2020.0191 34365915

[B53] Page A. J. Cummins C. A. Hunt M. Wong V. K. Reuter S. Holden M. T. (2015). Roary: rapid large-scale prokaryote pan genome analysis. *Bioinformatics* 31 3691–3693. 10.1093/bioinformatics/btv421 26198102 PMC4817141

[B54] Pagnini C. Corleto V. D. Martorelli M. Lanini C. D’Ambra G. Di Giulio E. (2018). Mucosal adhesion and anti-inflammatory effects of Lactobacillus rhamnosus GG in the human colonic mucosa: A proof-of-concept study. *World J. Gastroenterol.* 24 4652–4662. 10.3748/wjg.v24.i41.4652 30416313 PMC6224475

[B55] Pavli F. G. Argyri A. A. Papadopoulou O. S. Nychas J. G. E. Chorianopoulos N. G. Tassou C. C. (2016). Probiotic potential of lactic acid bacteria from traditional fermented dairy and meat products: assessment by in vitro tests and molecular characterization. *J. Prob. Health* 4:1000157. 10.4172/2329-8901.1000157

[B56] Plessas S. Kiousi D. E. Rathosi M. Alexopoulos A. Kourkoutas Y. Mantzourani I. (2020). Isolation of a *Lactobacillus paracasei* strain with probiotic attributes from kefir grains. *Biomedicines* 8:594. 10.3390/biomedicines8120594 PMC7764135 33322295

[B57] Pritchard L. Glover R. H. Humphris S. Elphinstone J. G. Toth I. K. (2016). Genomics and taxonomy in diagnostics for food security: Soft-rotting enterobacterial plant pathogens. *Anal. Methods* 8 12–24. 10.1039/c5ay02550h

[B58] Reunanen J. von Ossowski I. Hendrickx A. P. Palva A. de Vos W. M. (2012). Characterization of the SpaCBA pilus fibers in the probiotic *Lactobacillus rhamnosus* GG. *Appl. Environ. Microbiol*. 78 2337–2344. 10.1128/AEM.07047-11 22247175 PMC3302623

[B59] Rodrigo-Torres L. Yépez A. Aznar R. Arahal D. R. (2019). Genomic insights into five strains of *Lactobacillus plantarum* with biotechnological potential isolated from chicha, a traditional maize-based fermented beverage from Northwestern Argentina. *Front. Microbiol.* 10:2232. 10.3389/fmicb.2019.02232 31611862 PMC6773835

[B60] Ruiz L. Margolles A. Sánchez B. (2013). Bile resistance mechanisms in *Lactobacillus* and *Bifidobacterium*. *Front. Microbiol.* 4:396. 10.3389/fmicb.2013.00396 24399996 PMC3872040

[B61] Shao Y. Zhang W. Guo H. Pan L. Zhang H. Sun T. (2015). Comparative studies on antibiotic resistance in *Lactobacillus casei* and *Lactobacillus plantarum*. *Food Control* 50 250–258. 10.1016/j.foodcont.2014.09.003

[B62] Sharma D. Saharan B. S. (2016). Functional characterization of biomedical potential of biosurfactant produced by *Lactobacillus helveticus*. *Biotechnol. Rep.* 11 27–35. 10.1016/j.btre.2016.05.001 28352537 PMC5042301

[B63] Siciliano R. A. Lippolis R. Mazzeo M. F. (2019). Proteomics for the investigation of surface-exposed proteins in probiotics. *Front. Nutr.* 6:52. 10.3389/fnut.2019.00052 31069232 PMC6491629

[B64] Siegel S. D. Reardon M. E. Ton-That H. (2017). Anchoring of LPXTG-like proteins to the gram-positive cell wall envelope. *Curr. Top. Microbiol. Immunol.* 404 159–175. 10.1007/82_2016_8 27097813

[B65] Tatusova T. DiCuccio M. Badretdin A. Chetvernin V. Nawrocki E. P. Zaslavsky L. (2016). NCBI prokaryotic genome annotation pipeline. *Nucleic Acids Res.* 44 6614–6624. 10.1093/nar/gkw569 27342282 PMC5001611

[B66] Teame T. Wang A. Xie M. Zhang Z. Yang Y. Ding Q. (2020). Paraprobiotics and postbiotics of probiotic *Lactobacilli*, Their Positive Effects on the Host and Action Mechanisms: A Review. *Front. Nutr.* 7:570344. 10.3389/fnut.2020.570344 33195367 PMC7642493

[B67] Thakur P. Gantasala N. P. Choudhary E. Singh N. Abdin M. Z. Agarwal N. (2016). The preprotein translocase YidC controls respiratory metabolism in *Mycobacterium tuberculosis*. *Sci. Rep.* 6:24998. 10.1038/srep24998 27166092 PMC4863248

[B68] Tian P. O’Riordan K. J. Lee Y. K. Wang G. Zhao J. Zhang H. (2020). Towards a psychobiotic therapy for depression: *Bifidobacterium breve* CCFM1025 reverses chronic stress-induced depressive symptoms and gut microbial abnormalities in mice. *Neurobiol. Stress* 12:100216. 10.1016/j.ynstr.2020.100216 32258258 PMC7109524

[B69] Tiptiri-Kourpeti A. Spyridopoulou K. Santarmaki V. Aindelis G. Tompoulidou E. Lamprianidou E. E. (2016). *Lactobacillus casei* exerts anti-proliferative effects accompanied by apoptotic cell death and Up-Regulation of TRAIL in colon carcinoma cells. *PLoS One* 11:e0147960. 10.1371/journal.pone.0147960 26849051 PMC4744000

[B70] Tuo Y. Song X. Song Y. Liu W. Tang Y. Gao Y. (2018). Screening probiotics from *Lactobacillus* strains according to their abilities to inhibit pathogen adhesion and induction of pro-inflammatory cytokine IL-8. *J. Dairy Sci.* 101 4822–4829. 10.3168/jds.2017-13654 29550135

[B71] Tuyarum C. Songsang A. Lertworapreecha M. (2021). *In vitro* evaluation of the probiotic potential of *Lactobacillus* isolated from native swine manure. *Vet. World* 14 1133–1142. 10.14202/vetworld.2021.1133-1142 34220114 PMC8243659

[B72] Tytgat H. L. Douillard F. P. Reunanen J. Rasinkangas P. Hendrickx A. P. Laine P. K. (2016). *Lactobacillus rhamnosus* GG outcompetes *Enterococcus faecium* via Mucus-Binding Pili: evidence for a novel and heterospecific probiotic mechanism. *Appl. Environ. Microbiol.* 82 5756–5762. 10.1128/AEM.01243-16 27422834 PMC5038030

[B73] van den Nieuwboer M. van Hemert S. Claassen E. de Vos W. M. (2016). *Lactobacillus plantarum* WCFS1 and its host interaction: a dozen years after the genome. *Microb. Biotechnol*. 9 452–465. 10.1111/1751-7915.12368 27231133 PMC4919987

[B74] Walsham A. D. MacKenzie D. A. Cook V. Wemyss-Holden S. Hews C. L. Juge N. (2016). *Lactobacillus reuteri* inhibition of enteropathogenic *Escherichia coli* adherence to human intestinal epithelium. *Front. Microbiol.* 7:244. 10.3389/fmicb.2016.00244 26973622 PMC4771767

[B75] Winter J. Linke K. Jatzek A. Jakob U. (2005). Severe oxidative stress causes inactivation of DnaK and activation of the redox-regulated chaperone Hsp33. *Mol. Cell.* 17 381–392. 10.1016/j.molcel.2004.12.027 15694339

[B76] Wu Y. Wang B. Xu H. Tang L. Li Y. Gong L. (2019). Probiotic *Bacillus* attenuates oxidative stress- induced intestinal injury via p38-mediated autophagy. *Front. Microbiol.* 10:2185. 10.3389/fmicb.2019.02185 31632359 PMC6779063

[B77] Ye K. Li P. Gu Q. (2020). Complete genome sequence analysis of a strain *Lactobacillus pentosus* ZFM94 and its probiotic characteristics. *Genomics* 112 3142–3149. 10.1016/j.ygeno.2020.05.015 32450257

[B78] Yu H. J. Chen Y. F. Yang H. J. Yang J. Xue J. G. Li C. K. (2015). Screening for *Lactobacillus plantarum* with potential inhibitory activity against enteric pathogens. *Ann. Microbiol.* 65 1257–1265. 10.1007/s13213-014-0963-3

[B79] Yu Z. Lavèn M. Klepsch M. de Gier J. W. Bitter W. van Ulsen P. (2011). Role for *Escherichia coli* YidD in membrane protein insertion. *J. Bacteriol.* 193 5242–5251. 10.1128/JB.05429-11 21803992 PMC3187451

[B80] Zheng J. Ruan L. Sun M. Gänzle M. A. (2015). Genomic view of *Lactobacilli* and *Pediococci* demonstrates that phylogeny matches ecology and physiology. *Appl. Environ. Microbiol.* 81 7233–7243. 10.1128/AEM.02116-15 26253671 PMC4579461

[B81] Zheng J. Wittouck S. Salvetti E. Franz C. M. A. P. Harris H. M. B. Mattarelli P. (2020). A taxonomic note on the genus *Lactobacillus*: description of 23 novel genera, emended description of the genus *Lactobacillus* Beijerinck 1901, and union of *Lactobacillaceae* and *Leuconostocaceae*. *Int. J. Syst. Evol. Microbiol.* 70 2782–2858. 10.1099/ijsem.0.004107 32293557

