# Supplementary material for: Whole-Genome Sequencing, Phylogenetic and Genomic Analysis of Lactiplantibacillus pentosus L33, a Potential Probiotic Strain Isolated From Fermented Sausages
Source: Front Microbiol. 2021 Oct 26;12:746659. doi: 10.3389/fmicb.2021.746659 (PMC8576124; doi:10.3389/fmicb.2021.746659)
Supplement: Supplementary file 1 [file Data_Sheet_1.zip › Data Sheet 1/Supplementary Figure 1.PDF]

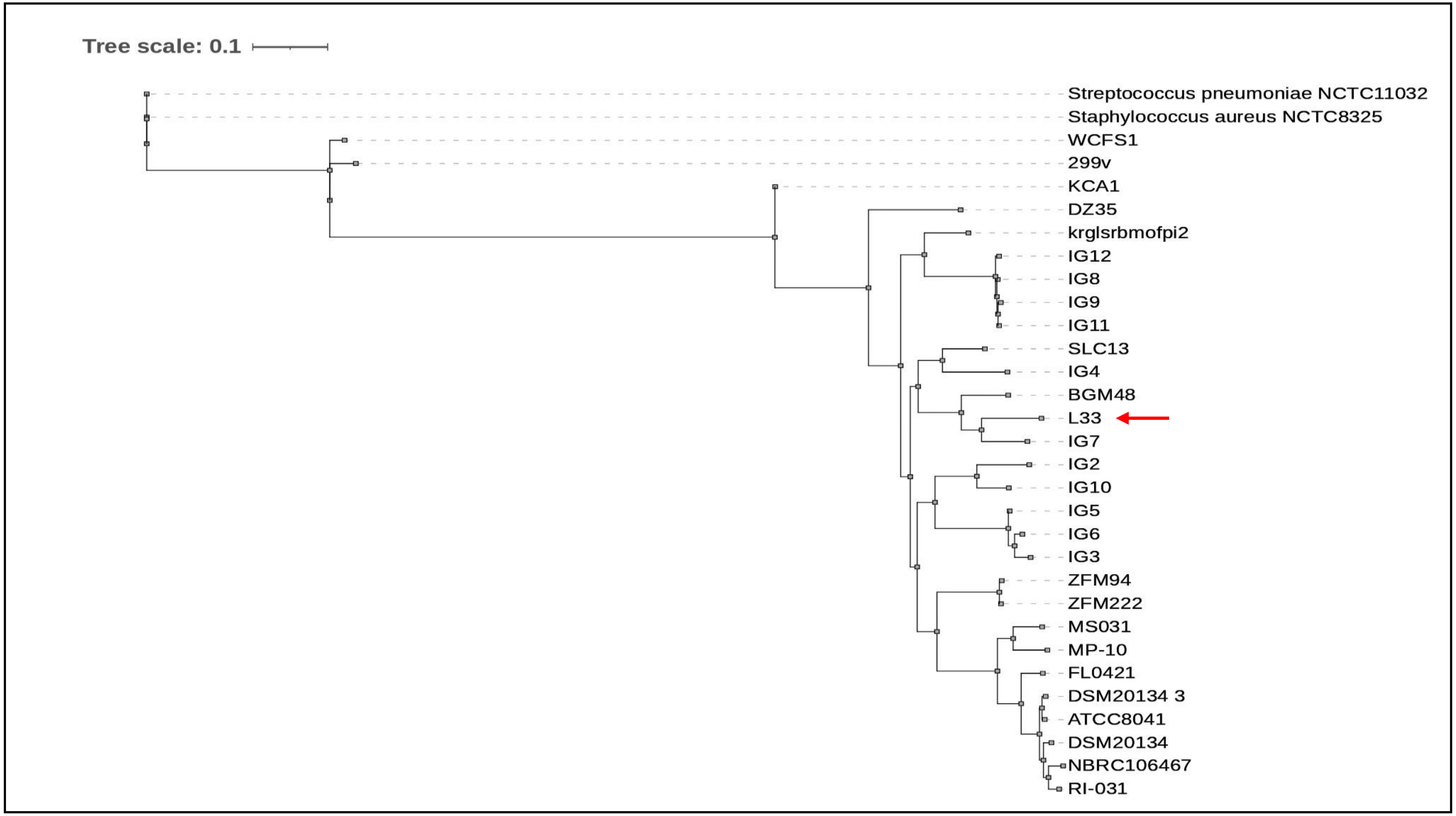

Supplementary Figure 1: Neighbor-joining phylogenetic tree based on orthologous genes found by Roary (version 3.13.0), of *L. pentosus* L33, 26 *L. pentosus* strains and 2 probiotic *L. plantarum* strains; *L. plantarum WCFS1 and L. plantarum* 299v. *Streptococcus pneumoniae* NCTC11032 and *Staphylococcus aureus* NCTC8325 have been used as outgroups/controls.
