# Supplementary material for: Whole-Genome Sequencing, Phylogenetic and Genomic Analysis of Lactiplantibacillus pentosus L33, a Potential Probiotic Strain Isolated From Fermented Sausages
Source: Front Microbiol. 2021 Oct 26;12:746659. doi: 10.3389/fmicb.2021.746659 (PMC8576124; doi:10.3389/fmicb.2021.746659)
Supplement: Supplementary file 1 [file Data_Sheet_1.zip › Data Sheet 1/Supplementary Figure 2.PDF]

```
L33-b peptide: MNKLNDFYILNEGNLSQIEGGSG----FSLGLEILLSAYKHRKTIEKSFNKGFYN
               MN LN F  L + +LSQIEGGS     ++LG++IL SAYKHRKTIEKSFNKGFY+
NC8-β peptide: MNNLNKFSTLGKSSLSQIEGGSVPTSVYTLGIKILWSAYKHRKTIEKSFNKGFYH


L33-a peptide:  MKFQTISASNLATITGGDLTTKLWSSWGYYLGKKARWNLKHPYVQF
                 KF+ IS SNL  I+GGDLTTKLWSSWGYYLGKKARWNLKHPYVQF
NC8-α peptide: MDKFEKISTSNLEKISGGDLTTKLWSSWGYYLGKKARWNLKHPYVQF
```

Supplementary Figure 2: Amino acid sequences alignment of the 2 peptides that form the *L. pentosus* L33 class IIb bacteriocin against their homologous peptides of the plantaricin NC8 αβ (WP_003643800.1, AAO18426.1). GxxxG and GxxxG-like motifs are highlighted with yellow. Red color shows the missing SxxxS motif, in L33. The lack of this motif possibly affects the bacteriocin's polymerization and consequently its functionality.
