# Supplementary material for: Whole-Genome Sequencing, Phylogenetic and Genomic Analysis of Lactiplantibacillus pentosus L33, a Potential Probiotic Strain Isolated From Fermented Sausages
Source: Front Microbiol. 2021 Oct 26;12:746659. doi: 10.3389/fmicb.2021.746659 (PMC8576124; doi:10.3389/fmicb.2021.746659)
Supplement: Supplementary file 1 [file Data_Sheet_1.zip › Data Sheet 1/Supplementary Figure 6.PDF]

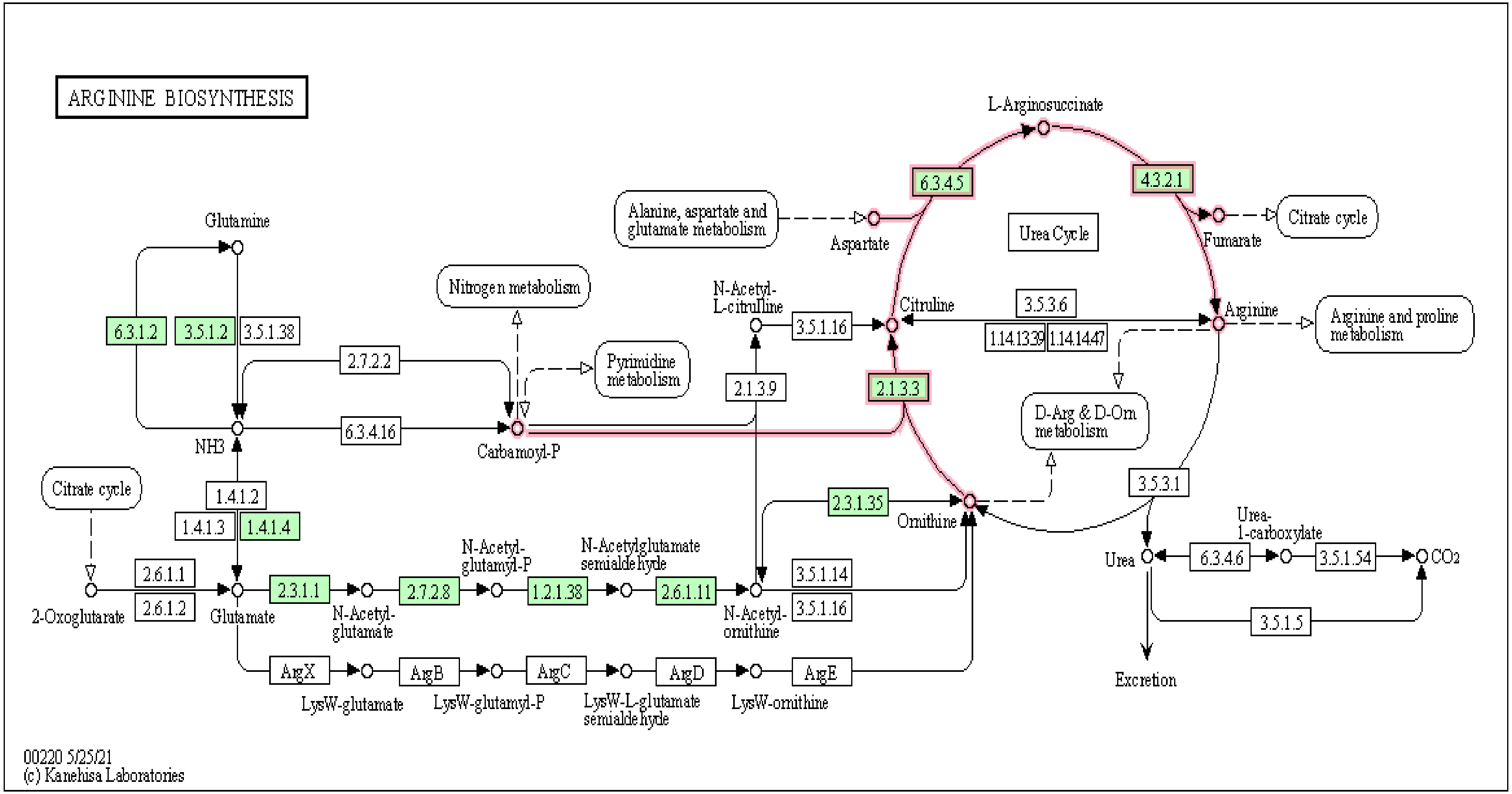

Supplementary Figure 6: Arginine Metabolism KEGG pathway (ko: 00220) as constructed by KEGG pathway reconstruction tool. Each box represents a protein involved in the pathway. Proteins in green colored boxes are present in *L. pentosus* L33. Green boxes outlined with pink color make up the complete arginine biosynthesis module (M00844), indicating the capability of *L. pentosus* L33 to produce Arginine.
