# Supplementary material for: Whole-Genome Sequencing, Phylogenetic and Genomic Analysis of Lactiplantibacillus pentosus L33, a Potential Probiotic Strain Isolated From Fermented Sausages
Source: Front Microbiol. 2021 Oct 26;12:746659. doi: 10.3389/fmicb.2021.746659 (PMC8576124; doi:10.3389/fmicb.2021.746659)
Supplement: Supplementary file 1 [file Data_Sheet_1.zip › Data Sheet 1/Supplementary Table 1.PDF]

Supplementary Table 1. The number of tRNAs corresponding to the 20 amino acids encoded by *L. pentosus* L33 predicted by the NCBI Prokaryotic Genome Annotation Pipeline (PGAP) algorithm.

Amino-acids	Number of tRNA genes	Gene ID	Gene ID2	Gene ID3	Gene ID4	Gene ID5
Ala	3	L33_000086	L33_000145	L33_001098	-	-
Arg	3	L33_001780	L33_002709	L33_001975	-	-
Asn	4	L33_003208	L33_000089	L33_001888	L33_003022	-
Asp	2	L33_000134	L33_001195	-	-	-
Cys	1	L33_000098	-	-	-	-
Glu	3	L33_000092	L33_000136	L33_001630	-	-
Gln	3	L33_000097	L33_001632	L33_002450	-	-
Gly	5	L33_000139	L33_000231	L33_001196	L33_002151	L33_003039
His	2	L33_000096	L33_001534	-	-	-
Ile	5	L33_000138	L33_000142	L33_001601	L33_002150	L33_002823
Leu	5	L33_000099	L33_000230	L33_000233	L33_001537	L33_002484
Lys	1	L33_000563	-	-	-	-
Met	3	L33_000135	L33_000140	L33_000143	-	-
Phe	1	L33_000093	-	-	-	-
Pro	1	L33_000229	-	-	-	-
Ser	4	L33_000090	L33_000137	L33_000141	L33_002798	-
Thr	3	L33_000232	L33_002391	L33_002446	-	-
Trp	2	L33_000095	L33_001600	-	-	-
Tyr	2	L33_000094	L33_002449	-	-	-
Val	1	L33_000092	-	-	-	-
Other	4	L33_003041	L33_003073	L33_003228	L33_003229	-
