# Supplementary material for: Whole-Genome Sequencing, Phylogenetic and Genomic Analysis of Lactiplantibacillus pentosus L33, a Potential Probiotic Strain Isolated From Fermented Sausages
Source: Front Microbiol. 2021 Oct 26;12:746659. doi: 10.3389/fmicb.2021.746659 (PMC8576124; doi:10.3389/fmicb.2021.746659)
Supplement: Supplementary file 1 [file Data_Sheet_1.zip › Data Sheet 1/Supplementary Table 2.PDF]

Supplementary Table 2. Clustered regularly interspaced palindromic repeats (CRISPR) arrays detected in the genome of *L. pentosus* L33 by CRISPRDetect (version 2.4).

Name	Array Position	Type	Direct Repeats	Direct Repeats Sequence	Direct Repeats Length (bp)	Spacer Sequence Average Length (bp)
CRISPR_1	scaffold2|size236308:76258-75803	I-E	8	CTGTTCCCCGCGTATGCGGGGGTGATCC	28	33
CRISPR_2	scaffold2|size236308:79569-78877	I-E	12	CTGTTCCCCGTGTATGCGGGGGTGATCC	28	32
CRISPR_3	scaffold15|size90719:6371-5375	II-A	15	GTCTTGAATAGTAGTCATATCAAACAGGTTTAGAAC	36	33
