# Supplementary material for: Whole-Genome Sequencing, Phylogenetic and Genomic Analysis of Lactiplantibacillus pentosus L33, a Potential Probiotic Strain Isolated From Fermented Sausages
Source: Front Microbiol. 2021 Oct 26;12:746659. doi: 10.3389/fmicb.2021.746659 (PMC8576124; doi:10.3389/fmicb.2021.746659)
Supplement: Supplementary file 1 [file Data_Sheet_1.zip › Data Sheet 1/Supplementary Table 3.PDF]

**Supplementary Table 3.** Putative prophage sequences annotated in genome assembly of *L. pentosus* L33 by PHAge Search Tool Enhanced Release (PHASTER).

| Region Position | Total tRNAs | Phage Proteins | Hypothetical Proteins | Bacterial Proteins | Total Proteins | GC(%) | Most Common Phage | Accession |
|---|---|---|---|---|---|---|---|---|
| scaffold4\|size168663:1112-30720 | 0 | 23 | 13 | 0 | 36 | 41,89 | *Listeria* phage vB_LmoS_293 | NC_028929 |
| scaffold29\|size47413:202-39852 | 4 | 35 | 20 | 0 | 55 | 42,62 | *Oenococcus* phage phiS13 | NC_023560 |
| scaffold42\|size25001:64-24462 | 0 | 28 | 9 | 0 | 37 | 40,95 | *Lactobacillus* phage Sha1 | NC_019489 |
| scaffold46\|size22098:867-19673 | 0 | 17 | 3 | 0 | 20 | 40,84 | *Lactobacillus* phage phiAT3 | NC_005893 |
