# Supplementary material for: Whole-Genome Sequencing, Phylogenetic and Genomic Analysis of Lactiplantibacillus pentosus L33, a Potential Probiotic Strain Isolated From Fermented Sausages
Source: Front Microbiol. 2021 Oct 26;12:746659. doi: 10.3389/fmicb.2021.746659 (PMC8576124; doi:10.3389/fmicb.2021.746659)
Supplement: Supplementary file 1 [file Data_Sheet_1.zip › Data Sheet 1/Supplementary Table 7.PDF]

**Supplementary Table 7:** List of complete KEGG modules and pathways that reflect the amino acid biosynthesis capability of *L. pentosus* L33.

| Amino acid | KEGG Pathway | KEGG Module | Status | Number of present Genes in the Biosynthetic pathway/module |
|---|---|---|---|---|
| **Thr** | ko00260 | M00018 | Complete | 5 out of 5 |
| **Cys** | ko00270 | M00021 | Complete | 2 out of 2 |
| **Met** | ko00270 | M00017 | Complete | 7 out of 7 |
| **Lys** | ko00300 | M00525 | Complete | 9 out of 9 |
| **Arg** | ko00220 | M00844 | Complete | 3 out of 3 |
| **Pro** | ko00330 | M00015 | Complete | 2 out of 2 |
| **His** | ko00340 | M00026 | Complete | 6 out of 6 |
