# Supplementary figures and images for: Whole-Genome Sequencing, Phylogenetic and Genomic Analysis of Lactiplantibacillus pentosus L33, a Potential Probiotic Strain Isolated From Fermented Sausages
Source: Front Microbiol. 2021 Oct 26;12:746659. doi: 10.3389/fmicb.2021.746659 (PMC8576124; doi:10.3389/fmicb.2021.746659)

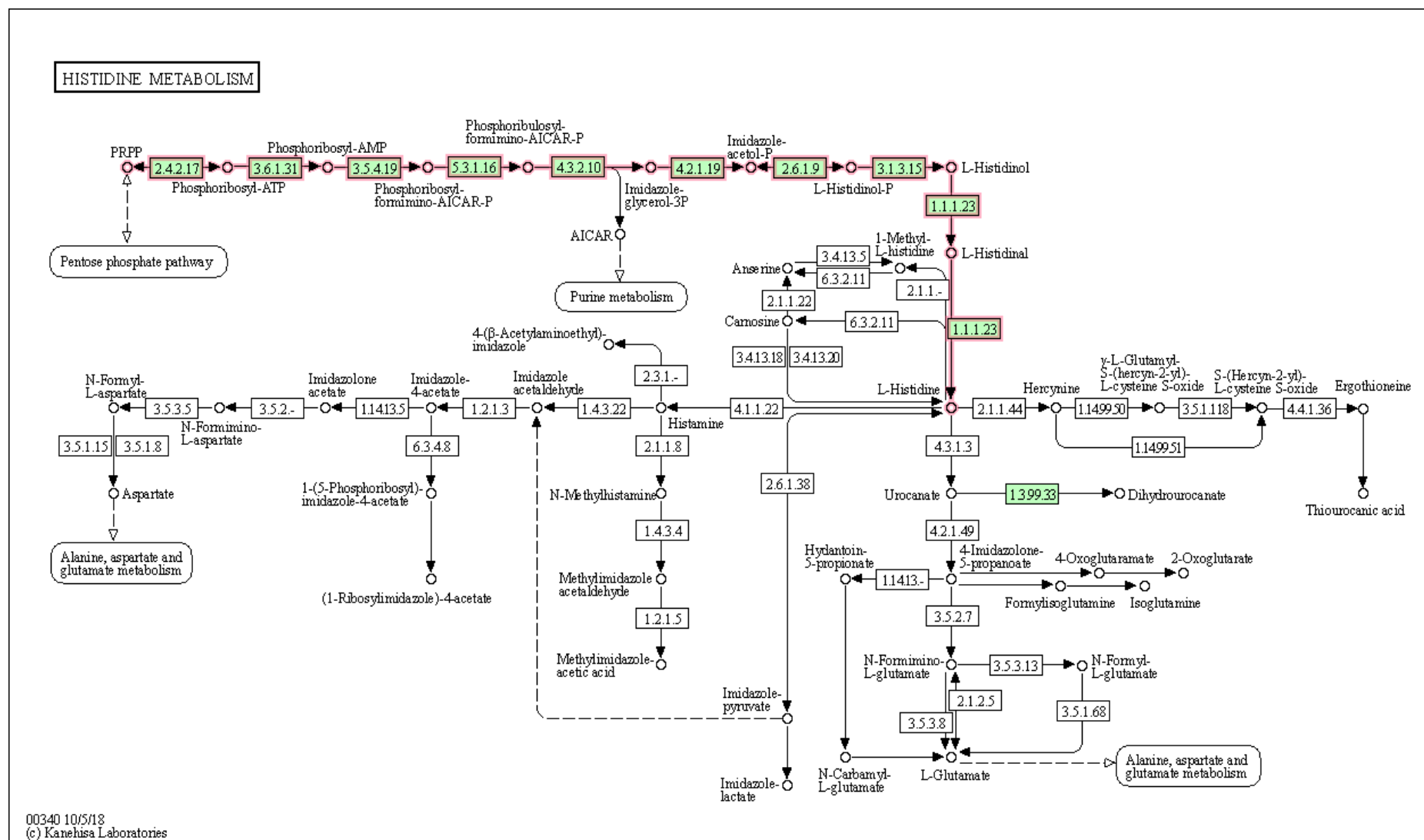

Supplement: Supplementary file 1 [file Data_Sheet_1.zip › Data Sheet 1/Supplementary Figure 8.PDF]
